# Activation of Ca^2+^-dependent proteolysis in skeletal muscle and heart in cancer cachexia

**DOI:** 10.1054/bjoc.2001.1696

**Published:** 2001-04

**Authors:** P Costelli, R De Tullio, F M Baccino, E Melloni

**Affiliations:** Dipartimento di Medicina ed Oncologia Sperimentale, Sezione di Patologia Generale, Università di Torino; Dipartimento di Medicina Sperimentale, Sezione di Biochimica, Università di Genova; Centro CNR di Immunogenetica ed Oncologia Sperimentale, Torino, Italy

**Keywords:** calpain, protein breakdown, muscle wasting

## Abstract

Cachexia is a syndrome characterized by profound tissue wasting that frequently complicates malignancies. In a cancer cachexia model we have shown that protein depletion in the skeletal muscle, which is a prominent feature of the syndrome, is mostly due to enhanced proteolysis. There is consensus on the views that the ubiquitin/proteasome pathway plays an important role in such metabolic response and that cytotoxic cytokines such as TNFα are involved in its triggering (Costelli and Baccino, 2000), yet the mechanisms by which the relevant extracellular signals are transduced into protein hypercatabolism are largely unknown. Moreover, little information is presently available as to the possible involvement in muscle protein waste of the Ca^2+^-dependent proteolysis, which may provide a rapidly activated system in response to the extracellular signals. In the present work we have evaluated the status of the Ca^2+^-dependent proteolytic system in the gastrocnemius muscle of AH-130 tumour-bearing rats by assaying the activity of calpain as well as the levels of calpastatin, the natural calpain inhibitor, and of the 130 kDa Ca^2+^-ATPase, both of which are known calpain substrates. After tumour transplantation, total calpastatin activity progressively declined, while total calpain activity remained unchanged, resulting in a progressively increasing unbalance in the calpain/calpastatin ratio. A decrease was also observed for the 130 kDa plasma membrane form of Ca^2+^-ATPase, while there was no change in the level of the 90 kDa sarcoplasmic Ca^2+^-ATPase, which is resistant to the action of calpain. Decreased levels of both calpastatin and 130 kDa Ca^2+^-ATPase have been also detected in the heart of the tumour-bearers. These observations strongly suggest that Ca^2+^-dependent proteolysis was activated in the skeletal muscle and heart of tumour-bearing animals and raise the possibility that such activation may play a role in sparking off the muscle protein hypercatabolic response that characterizes cancer cachexia. © 2001 Cancer Research Campaignhttp://www.bjcancer.com


*British Journal of Cancer* (2001) **84**(7), 946–950
© 2001 Cancer Research Campaign
doi: 10.1054/ bjoc.2001.1696, available online at http://www.idealibrary.com on IDEAL
http://www.bjcancer.com

### Activation of $Ca^{2+}$-dependent proteolysis in skeletal muscle and heart in cancer cachexia

**P Costelli[1], R De Tullio[2], FM Baccino[1,3] and E Melloni[2]**

[1]Dipartimento di Medicina ed Oncologia Sperimentale, Sezione di Patologia Generale, Università di Torino, [2]Dipartimento di Medicina Sperimentale, Sezione di Biochimica, Università di Genova, [3]Centro CNR di Immunogenetica ed Oncologia Sperimentale, Torino, Italy

**Summary** Cachexia is a syndrome characterized by profound tissue wasting that frequently complicates malignancies. In a cancer cachexia model we have shown that protein depletion in the skeletal muscle, which is a prominent feature of the syndrome, is mostly due to enhanced proteolysis. There is consensus on the views that the ubiquitin/proteasome pathway plays an important role in such metabolic response and that cytotoxic cytokines such as TNF$\alpha$ are involved in its triggering (Costelli and Baccino, 2000), yet the mechanisms by which the relevant extracellular signals are transduced into protein hypercatabolism are largely unknown. Moreover, little information is presently available as to the possible involvement in muscle protein waste of the $Ca^{2+}$-dependent proteolysis, which may provide a rapidly activated system in response to the extracellular signals. In the present work we have evaluated the status of the $Ca^{2+}$-dependent proteolytic system in the gastrocnemius muscle of AH-130 tumour-bearing rats by assaying the activity of calpain as well as the levels of calpastatin, the natural calpain inhibitor, and of the 130 kDa $Ca^{2+}$-ATPase, both of which are known calpain substrates. After tumour transplantation, total calpastatin activity progressively declined, while total calpain activity remained unchanged, resulting in a progressively increasing unbalance in the calpain/calpastatin ratio. A decrease was also observed for the 130 kDa plasma membrane form of $Ca^{2+}$-ATPase, while there was no change in the level of the 90 kDa sarcoplasmic $Ca^{2+}$-ATPase, which is resistant to the action of calpain. Decreased levels of both calpastatin and 130 kDa $Ca^{2+}$-ATPase have been also detected in the heart of the tumour-bearers. These observations strongly suggest that $Ca^{2+}$-dependent proteolysis was activated in the skeletal muscle and heart of tumour-bearing animals and raise the possibility that such activation may play a role in sparking off the muscle protein hypercatabolic response that characterizes cancer cachexia. © 2001 Cancer Research Campaign http://www.bjcancer.com

**Keywords**: calpain; protein breakdown; muscle wasting

Cancer cachexia, a syndrome characterized by profound body wasting due to a persistently negative nitrogen balance, is an important cause of death among cancer patients and still defies an effective management (Calman, 1992; Tisdale, 1997). Different mechanisms, often in combination, are involved in its pathogenesis, including metabolic competition for nutrients between tumour and host tissues or protein-calorie malnutrition, but a prominent role is played by circulating mediators and hormonal perturbations (Tessitore et al, 1993a; Soeter and Baracos, 1999; Costelli and Baccino, 2000). The net protein loss is particularly conspicuous in the skeletal muscle and heart and has been shown to result from enhanced protein degradation in various clinical or experimental situations (Kien and Camitta, 1983; Kien and Camitta, 1987; Tessitore et al, 1987; Melville et al, 1990; Beck et al, 1991; Tessitore et al, 1993b; Temparis et al, 1994; Baracos et al, 1995).

The machinery involved in this muscle protein waste has not been fully elucidated yet. Not only the precise role of the different proteolytic systems still has to be assessed, but there is little information as to how the catabolic signals are transduced into enhanced tissue protein breakdown. There is evidence that the ATP-ubiquitin-dependent pathway plays a major role in the accelerated muscle proteolysis, both in experimental models of cancer cachexia and in cancer patients (Temparis et al, 1994; Baracos et al, 1995; Llovera et al, 1995; Williams et al, 1999a; Bossola M et al, unpublished observations), although how activation of this proteolytic system is triggered is not known. By contrast, in spite of reports of increased lysosomal cathepsin levels (Lundholm et al, 1980; Tessitore et al, 1993b), there is evidence to deny a role for the lysosomal proteolytic pathway, since protein breakdown in muscles isolated from tumour-bearing rats was not affected by the use of appropriate inhibitors (Temparis et al, 1994; Baracos et al, 1995; Llovera et al, 1995).

As to the $Ca^{2+}$-dependent proteolytic system, little information is available at present. On one hand, m-calpain mRNA was found increased in the muscles of AH-130 Yoshida tumour-bearing rats (Temparis et al, 1994). On the other, decreasing the intracellular $Ca^{2+}$ concentration ($[Ca^{2+}]_i$) or adding thiol proteinase inhibitors did not affect overall protein degradation rates in muscle preparations from these animals (Temparis et al, 1994; Baracos et al, 1995; Llovera et al, 1995). However, since the $Ca^{2+}$-dependent proteolytic system is thought to be involved in the cleavage of specific protein targets rather than in bulk protein degradation the latter observations do not necessarily rule out an activation of the calpain system.

A general difficulty encountered in studies on $Ca^{2+}$-dependent proteolysis is that calpain activity assays do not provide a measure of the actual enzyme activity in vivo. Nor are the levels of calpain mRNA or protein informative in this regard. In such studies, however, advantage can be taken of the fact that calpain activation in vivo directly results in cleavage of specific intracellular substrate proteins (Salamino et al, 1994a; Nath et al, 1996;

*Received 7 August 2000*
*Accepted 15 January 2001*

*Correspondence to:* P Costelli

Carafoli and Molinari, 1998). Measuring the time-course of changes in substrate levels may thus provide an estimate of the calpain activity in vivo cumulated over time. In the present work we adopted this approach and selected two independent reporter substrates, namely, calpastatin, the endogenous calpain inhibitor that is cleaved by calpain itself (Pontremoli et al, 1991), and the 130 kDa $Ca^{2+}$-ATPase associated with the plasma membrane (Salamino et al, 1992, 1994b). As an internal control for the latter substrate, we also measured the levels of the sarcoplasmic 90 kDa ATPase, which is resistant to proteolysis by calpain (Yoshida et al, 1990). Moreover, we also assayed tissues for calpain activity in vitro, thus obtaining an evaluation of the actual enzyme levels as well as of the calpain/calpastatin balance.

The data show that both calpastatin and 130 kDa $Ca^{2+}$-ATPase levels decreased in the gastrocnemius and heart of rats soon after implantation of the AH-130 Yoshida ascites hepatoma, while calpain activity did not significantly change. Loss of calpain substrates and increased calpain/calpastatin ratio both are strongly suggestive for an activation of $Ca^{2+}$-dependent proteolysis in these tissues.

### MATERIALS AND METHODS

The experiments were performed on male Wistar rats weighing about 150 g (Charles River, Como, Italy), housed and cared for in compliance with the Italian Ministry of Health Guidelines and with the Principles of Laboratory Animal Care (NIH n. 85–23, revised 1985). The animals were fed ad libitum on a chow diet (Piccioni, Brescia, Italy) and had free access to drinking water. They were divided into 2 groups, namely controls and tumour bearers: the latter received an intraperitoneal injection of about $10^8$ Yoshida AH-130 ascites hepatoma cells in 2 ml of sterile 0.9% NaCl, the former a comparable volume of saline. The animals were sacrificed under light ether anaesthesia 2, 4 or 6 days after tumour transplantation. Gastrocnemius muscle and heart were rapidly excised, weighed, frozen in liquid nitrogen and stored at –80˚C.

### Isolation of calpastatin and calpain

Hearts or gastrocnemius muscles (4 g) were minced, suspended in 5 volumes (w/v) of cold 0.25 M sucrose containing 0.5 mM β-mercaptoethanol and 1 mM EDTA, homogenized in a Waring Blendor homogenizer set at half-maximal speed (5 bursts of 15 s each), sonicated (4 bursts of 15 s each), and centrifuged at 100 000 ***g*** for 15 min. To isolate calpastatin, the supernatant was dialysed against 50 mM Na acetate/acetic acid buffer, pH 6.7, containing 0.1 mM EDTA and 0.5 mM β-mercaptoethanol. The samples were then separately loaded on a DE32 column (1 × 10 cm) equilibrated in the dialysis buffer, and proteins eluted with 200 ml of a linear 0–0.35 M NaCl gradient (Pontremoli et al, 1991; Salamino et al, 1994a). To isolate calpain, the supernatant was dialysed against 50 mM Na borate/boric acid buffer, pH 7.5, containing 0.1 mM EDTA, and 0.5 mM β-mercaptoethanol. NaCl was then added to a 0.3 M final concentration and the samples separately loaded on a Phenyl Sepharose CL-4B column (1 × 4 cm) equilibrated in the dialysis buffer with 0.3 M NaCl added. Calpain activity was eluted with 20 ml of the same buffer without NaCl in 1 ml fractions (Pontremoli et al, 1991; Salamino et al 1994).

### Calpain, calpastatin, and $Ca^{2+}$-ATPase activities

Calpastatin was assayed on aliquots (150 µl) of the fractions eluted from the DE32 column, heated at 90˚C for 3 min in order to inactivate any endogenous protease activity. The activity of calpastatin was determined using human erythrocyte calpain, the calpain isoform most sensitive to calpastatin inhibition, and using acid-denaturated bovine globin as substrate (Salamino et al, 1994a). By ion-exchange chromatography, calpastatin is resolved into 2 activity peaks that have been previously identified as due to 2 forms generated by post-translational modifications of the same molecule and named calpastatins I and II according to the elution order: the first is a non-phosphorylated form that is more active on µ-calpain, the second a phosphorylated form more active on m-calpain (Pontremoli et al, 1992). Since the changes reproduced precisely the same pattern for both calpastatins (not shown), data are presented as the sum of their activities.

Calpain activity was assayed on aliquots (100 µl) of the fractions eluted from the Phenyl Sepharose column. One unit of calpastatin activity is the amount required to inhibit one unit of calpain, the latter being defined as the amount that releases 1 µmol $h^{-1}$ of free α-amino groups under the conditions specified (Salamino et al, 1994a).

$Ca^{2+}$-ATPase activity in cell membranes isolated from heart or gastrocnemius muscle was determined as previously reported (Niggli et al, 1979). After SDS-polyacrylamide gel electrophoresis and autoradiography, 2 bands were detected: a protein with an Mr of about 130 kDa, identified as the plasma membrane $Ca^{2+}$-ATPase (De Smedt et al, 1991), and a second band with an $M_r$ of about 90 kDa corresponding to the sarcoplasmic reticulum $Ca^{2+}$-ATPase (Yoshida et al, 1990).

### RESULTS

Since day 2 after inoculation into rats, the Yoshida AH-130 ascites hepatoma elicited a rapid and progressive weight loss in both the gastrocnemius muscle and heart, down to 60% of controls (Figure 1), as previously reported (Tessitore et al, 1987, 1993b). We analysed these tissues for parameters adequate to provide an evaluation of the status of $Ca^{2+}$-dependent proteolysis, as discussed earlier. The activity of calpains reflects the balance between enzyme levels and levels of calpastatins, their specific natural inhibitors

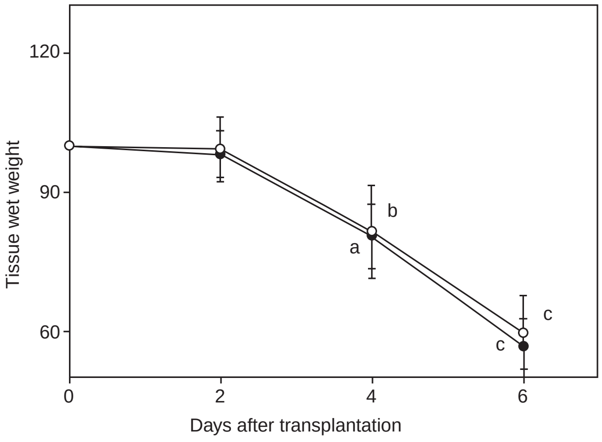

**Figure 1** Gastrocnemius muscle (●) and heart (O) wet weight. Data are expressed as percentages of control values. Significance of the differences: a = $P < 0.05$, b = $P < 0.01$, c = $P < 0.001$

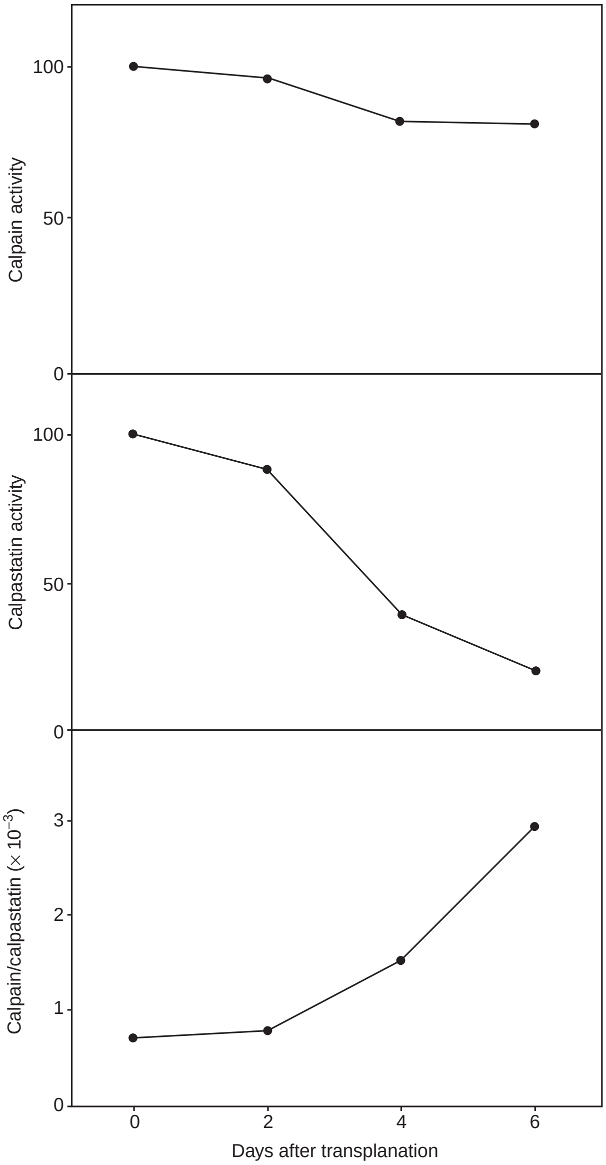

**Figure 2** Calpain and calpastatin activities and their ratio in gastrocnemius muscle. Data (U/total tissue) are expressed as percentages of controls. Calpain specific activity ranged from 0.171 U $mg^{-1}$ protein at day 0 to 0.216 U $mg^{-1}$ protein at day 6, capastatin specific activity from 239 U $mg^{-1}$ protein at day 0 to 74 U $mg^{-1}$ protein at day 6 after tumour transplantation

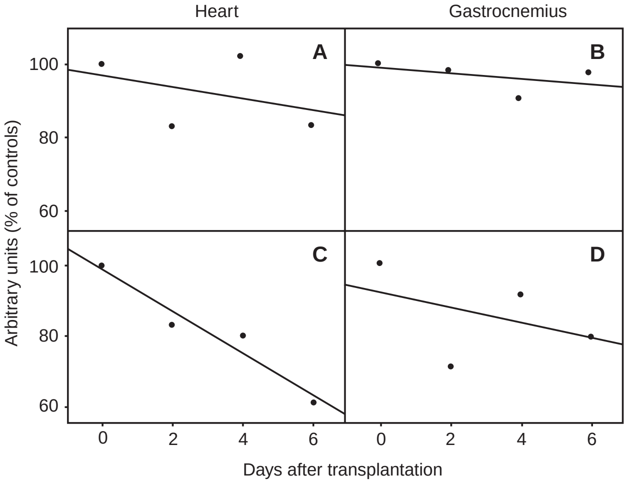

**Figure 3** Activities of 90 kDa $Ca^{2+}$-ATPase (**A, B**) and 130 kDa $Ca^{2+}$-ATPase (**C, D**) in the gastrocnemius muscle and heart. (**A**): r = 0.402, *P* = 0.598; (**B**): r = 0.540, *P* = 0.456; (**C**): r = 0.966, *P* = 0.034; (**D**): r = 0.428, *P* = 0.573

(Salamino et al, 1992). Activation of calpains proceeds from an increase of $[Ca^{2+}]_i$ through the cleavage of calpastatins, resulting in the release of active enzyme from the inactive complex. This basic regulatory mechanism permits the activity of the calpain system to be promptly tuned in response to manipulations or extracellular signals that elevate the $[Ca^{2+}]_i$. As a relevant example, decreased (50–90%) calpastatin levels have been observed in red blood cells from patients affected by essential hypertension (Pontremoli et al, 1988) or from genetically hypertensive rats of the Milan strain (Pontremoli et al, 1986); in these erythrocytes the plasma membrane $Ca^{2+}$-ATPase is very rapidly degraded in response to increases in $[Ca^{2+}]_i$. In the present work, total calpain and calpastatin activities were assayed in the gastrocnemius at increasing times after tumour implantation. As Figure 2 shows, while calpain activity remained virtually unchanged over the whole length of the experimental time, calpastatin activity showed a progressive, substantial decrease down to about 20% of the control value on day 6. Consequently, the calpain/calpastatin ratio became increasingly unbalanced, reaching a 4-fold elevation at day 6.

The above data at least indicate that in the gastrocnemius of tumour-bearing animals the setting of $Ca^{2+}$-dependent proteolytic system was being shifted in such a way as to favour its activity. Moreover, since calpastatin itself is a specific calpain substrate in vivo (Pontremoli et al, 1991), its decrease also suggests that in this muscle there was an ongoing activation of the calpain system. To substantiate this inference, in the muscle of tumour-bearing animals we also measured how changed the level of the 130 kDa plasma-membrane $Ca^{2+}$-ATPase, another yet unrelated calpain substrate, and compared it to the level of the sarcoplasmic 90 kDa $Ca^{2+}$-ATPase, which has been reported to be resistant to calpain proteolysis (Yoshida et al, 1990). The results in Figure 3 indicate that, while the levels of 90 kDa $Ca^{2+}$-ATPase virtually did not change in the gastrocnemius after tumour implantation, those of the 130 kDa form, in spite of some fluctuations, decreased down to 79% of the control values at day 6. Altogether, therefore, the present data consistently support the possibility that $Ca^{2+}$-dependent proteolysis was activated in the gastrocnemius soon after transplantation of the AH-130 ascites hepatoma in rats.

Similar results were obtained for the heart, although calpain activity was not assayed in this tissue. The activity levels of both calpain substrates, calpastatin (Figure 4) and 130 kDa $Ca^{2+}$-ATPase (Figure 3), progressively and substantially decreased in the heart of tumour-bearers, respectively down to 52% and 61% of control values at day 6, while those of the 90 kDa $Ca^{2+}$-ATPase did not change (Figure 3). Therefore, the inference that in these animals the $Ca^{2+}$-dependent proteolysis likely was activated also in the cardiac muscle appears justified.

### DISCUSSION

The present findings provide circumstantial yet quite compelling evidence that, in the present model, the calpain system became activated in both the skeletal and the cardiac muscles of

© 2001 Cancer Research Campaign

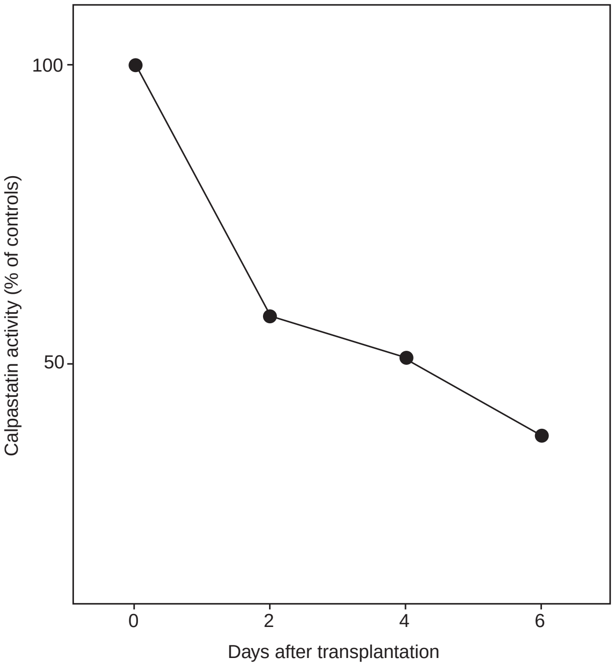

**Figure 4** Calpastatin activity in the heart. Data (U/total tissue) are expressed as percentages of control values tumour-bearing animals early during the development of cachexia. This conclusion is supported by 2 basic observations: (i) the marked, progressive elevation, in the gastrocnemius at least, of the calpain/calpastatin ratio which concurs in determining the pace of $Ca^{2+}$-dependent proteolysis in vivo; (ii) the progressive decrease in the activity levels of two specific calpain substrates such as calpastatin itself and the 130 kDa plasma-membrane $Ca^{2+}$-ATPase, which likely is the result of calpain activation. Noteworthily, decreased levels of the 130 kDa $Ca^{2+}$-ATPase may contribute to the maintenance of a $[Ca^{2+}]_i$ elevation (Vezzoli et al, 1985) and thus to calpain activation.

The increase of proteolytic rates in muscle preparations from tumour-bearing animals has been previously reported (Temparis et al, 1994; Baracos et al, 1995; Llovera et al, 1995) to be suppressed neither by the addition of exogenous calpain inhibitors nor by decreasing the $[Ca^{2+}]_i$. This is not in conflict with the present conclusion since, by all available evidence, calpains do not contribute significantly to bulk protein degradation. Rather, the notion that calpains play a role in the activation of signal transduction pathways (Melloni et al, 1986; Hirai et al, 1991; Watt and Molloy, 1993; Liu et al, 1996) may provide useful perspectives for a better understanding of the mechanisms by which the extracellular signals acting on the muscle (Soeter and Baracos, 1999; Costelli and Baccino, 2000) drive the elevation of protein breakdown responsible for tissue waste in cancer cachexia. In this regard, we cannot exclude that activation of $Ca^{2+}$-dependent proteolysis might even precede bulk protein hypercatabolism and play a causative role in establishing this metabolic response. Although quite speculative so far, the latter hypothesis may be consistent with the report that the release of myofilament proteins from the skeletal muscle in sepsis is likely associated with activation of $Ca^{2+}$-dependent proteolysis (Williams et al, 1999b), while activation of the ATP-ubiquitin-dependent proteolytic system would be the consequence of an increased availability of substrates rather than the primary cause of muscle protein breakdown (Williams et al, 1999b). Finally, the recent report (Busquets et al, 2000) that the expression of the muscle-specific calpain p94 is significantly reduced may also be relevant to the present findings. The loss of function of this particular calpain is responsible for limb girdle muscular dystrophy type 2A (Richard et al, 1995) and a suggested function of this enzyme is to protect some muscle proteins from degradation by ubiquitous m-and μ-calpains (Kinbara et al, 1998).

As a final remark, tumour necrosis factor (TNF) is an important mediator of cachexia in AH-130 tumour-bearing rats (Costelli et al, 1993; Tessitore et al, 1993a; Llovera et al, 1996) and in other situations. A reported effect of this cytokine, on some cell types at least, is to cause $[Ca^{2+}]_i$ elevations (Bick et al, 1997; Furukawa and Mattson, 1998), which, in the light of the present observations, might well have a role in activating the $Ca^{2+}$-dependent proteolysis. Furthermore, calpains have been shown to degrade transcription factors such as AP-1 and NF-κB (Hirai et al, 1991; Watt and Molloy, 1993; Liu et al, 1996) which are known to play a protective role against TNF-induced cell and tissue damage (Das et al, 1995; Jones et al, 1997); thus activation of the $Ca^{2+}$-dependent proteolysis might directly involve an attenuation of cell defences against the cytotoxic action of TNF. Therefore, the present observations provide new directions for future work aimed at elucidating the mechanisms underlying muscle protein waste in cancer cachexia.

### ACKNOWLEDGEMENTS

Work supported by Ministero per la Ricerca Scientifica e Tecnologica (MURST, Roma), Associazione Italiana per la Ricerca sul Cancro (Milano), Progetto Biotecnologie (MURST-CNR), MURST-PRIN 1997, Progetto Finalizzato Biotecnologie (CNR).

### REFERENCES

Baracos VE, De Vivo C, Hoyle DH and Goldberg AL (1995) Activation of the ATP-ubiquitin-proteasome pathway in skeletal muscle of cachectic rats bearing a hepatoma. *Am J Physiol* **268**: E996–E1006

Beck SA, Smith KL and Tisdale MJ (1991) Anticachectic and antitumour effect of eicosapentenoic acid and its effect on protein turnover. *Cancer Res* **51**: 6089–6093

Bick RJ, Liao JP, King TW, LeMaistre A, McMillin JB and Buja LM (1997) Temporal effects of cytokines on neonatal cardiac myocyte Ca2+ transients and adenylate cyclase activity. *Am J Physiol* **272**: H1937–H1944

Busquets S, Garcia-Martinez C, Alvarez B, Carbó N, López-Soriano FJ and Argilés JM (2000) Calpain-3 gene expression is decreased during experimental cancer cachexia. *Biochim Biophys Acta* **1475**: 5–9

Calman KC (1992) Cancer cachexia. In: *Oxford Textbook of Pathology*, McGee JO'D, Isaacson PG and Wright NA (eds) pp. 715–717. Oxford University Press: Oxford

Carafoli E and Molinari M (1998) Calpain: a protease in search of function? *Biochem Biophys Res Commun* **247**: 193–203

Costelli P and Baccino FM (2000) Cancer cachexia: from experimental models to patient management. *Curr Op Clin Nutr Metab Care* **3**: 177–181

Costelli P, Carbó N, Tessitore L, Bagby GJ, Lopez-Soriano FJ, Argilés JM and Baccino FM (1993) Tumor necrosis factor-alpha mediates changes in tissue protein turnover in a rat cancer cachexia model. *J Clin Invest* **92**: 2783–2789

Das KC, Lewis-Molock Y and White CW (1995) Activation of NF-kappa B and elevation of MnSOD gene expression by thiol reducing agents in lung adenocarcinoma (A549) cells. *Am J Physiol* **269**: L588–L602

De Smedt H, Eggermont JA, Wuytack F, Parys JB, Van Den Bosch L, Missiaen L, Verbis J and Casteels R (1991) Isoform switching of the sarco(endo)plasmic

© 2001 Cancer Research Campaign 

reticulum $Ca^{2+}$ pump during differentiation of BC3H1 myoblasts. *J Biol Chem* **266**: 7092–7095
Furukawa K and Mattson MP (1998) The transcription factor NF-kappaB mediates increases in calcium currents and decreases in NMDA-and AMPA/kainate-induced currents induced by tumor necrosis factor-alpha in hippocampal neurons. *J Neurochem* **70**: 1876–1886
Hirai S, Kawasaki H, Yaniv M and Suzuki K (1991) Degradation of transcription factors, c-Jun and c-Fos, by calpain. *FEBS Lett* **287**: 57–61
Jones PL, Ping D and Boss JM (1997) Tumor necrosis factor alpha and interleukin-1beta regulate the murine manganese superoxide dismutase gene through a complex intronic enhancer involving C/EBP-beta and NF-kappaB. *Mol Cell Biol* **17**: 6970–6981
Kien CL and Camitta BM (1983) Increased whole-body protein turnover in sick children with newly diagnosed leukemia or lymphoma. *Cancer Res* **43**: 5586–5592
Kien CL and Camitta BM (1987) Close association of accelerated rates of whole-body protein turnover (synthesis and breakdown) and energy expenditure in children with newly diagnosed acute lymphocytic leukemia. *J Parent Ent Nutr* **11**: 129–134
Kinbara K, Sorimachi H, Ishiura S and Suzuki K (1998) Skeletal muscle-specific calpain p94. Structure and function. *Biochem Pharmacol* **56**: 20106–20111
Liu Z.-Q, Kunimatsu M, Yang J-P, Ozaki Y, Sasaki M and Okamoto T (1996) Proteolytic processing of nuclear factor kB by calpain in vitro. *FEBS Lett* **385**: 109–113
Llovera M, Garcia-Martinez C, Agell N, Lopez-Soriano FJ and Argiles JM (1995) Muscle wasting associated with cancer cachexia is linked to an important activation of the ATP-dependent ubiquitin-mediated proteolysis. *Int J Cancer* **61**: 138–141
Llovera M, Carbó N, Garcia-Martinez C, Costelli P, Tessitore L, Baccino FM, Agell N, Bagby GJ, Lopez-Soriano FJ and Argiles JM (1996) Anti-TNF treatment reverts increased muscle ubiquitin gene expression in tumour-bearing rats. *Biochem Biophys Res Commun* **221**: 653–655
Lundholm K, Ekman L, Karlberg I, Edström S and Scherstèn T (1980) Comparison of hepatic cathepsin D activity in response to tumor growth and to caloric restriction in mice. *Cancer Res* **40**: 1680–1685
Melloni E, Pontremoli S, Michetti M, Sacco O, Sparatore B and Horecker BL (1986) The involvement of calpain in the activation of protein kinase C in neutrophils stimulated by phorbol myristic acid. *J Biol Chem* **61**: 4101–4105
Melville S, McNurlan MA, Graham-Calder A and Garlick PJ (1990) Increased protein turnover despite normal energy metabolism and responses to feeding in patients with lung cancer. *Cancer Res* **50**: 1125–1131
Nath R, Raser KJ, Stafford D, Hajikohammadreza I, Posner A, Allen H, Talanian RV, Yuen P, Gilbertsen RB and Wang KK (1996). *Biochem J* **319**: 683–690
Niggli V, Penniston JT and Carafoli E (1979) Purification of the (Ca2+-Mg2+)-ATPase from human erythrocyte membranes using a calmodulin affinity column. *J Biol Chem* **254**: 9955–9958
Pontremoli S, Melloni E, Salamino F, Sparatore B, Viotti PL, Michetti M, Duzzi L and Bianchi G (1986) Decreased level of calpain inhibitor activity in red blood cells from Milan hypertensive rats. *Biochem Biophys Res Commun* **138**: 1370–1375
Pontremoli S, Melloni E, Sparatore B, Pontremoli R, Tizianello A, Barlassina, Cusi D, Colombo R and Bianchi G (1988) Erythrocyte deficiency in calpain inhibitor activity in essential hypertension. *Hypertension* **12**: 474–479
Pontremoli S, Melloni E, Viotti PL, Michetti M, Salamino F and Horecker BL (1991) Identification of two calpastatin forms in rat skeletal muscle and their susceptibility to digestion by homologous calpains. *Arch Biochem Biophys* **288**: 646–652
Pontremoli S, Viotti PL, Michetti M, Salamino F, Sparatore B and Melloni E (1992) Modulation of inhibitory efficiency of rat skeletal muscle calpastatin by phosphorylation. *Biochem Biophys Res Commun* **187**: 751–759
Richard Y, Brous O, Allamand V, Fougerousse F, Chiannikulchai N, Bourg N, Bernguier L, Devaud C, Pasturaud P, Rodaut C, Hillaire D, Passos-Bueno M, Zatz M, Tishfield J, Fardeau M, Jackson C and Beckmann JS (1995) Mutations in the proteolytic enzyme calpain-3 cause limb-girdle muscular dystrophy type 2A. *Cell* **81**: 27–40
Salamino F, De Tullio R, Mengotti P, Viotti PL, Melloni E and Pontremoli S (1992) Different susceptibility of red cell membrane proteins to calpain degradation. *Arch Biochem Biophys* **298**: 287–292
Salamino F, De Tullio R, Michetti M, Mengotti P, Melloni E and Pontremoli S (1994a) Modulation of calpastatin specificity in rat tissues by reversible phosphorylation and dephosphorylation. *Biochem Biophys Res Commun* **199**: 1326–1332
Salamino F, Sparatore B, Melloni E, Michetti M, Viotti PL, Pontremoli S and Carafoli E (1994b) The plasma membrane calcium pump is the preferred calpain substrate within the erythrocyte. *Cell Calcium* **15**: 28–35
Soeter PB, Baracos VE (1999) Anabolic and catabolic mediators. *Curr Op Clin Nutr Metab Care* **2**: 195–199
Temparis S, Asensi M, Taillandier D, Aurousseau E, Larbaud D, Obled A, Bechet D, Ferrara M, Estrela JM and Attaix D (1994). Increased ATP-ubiquitin-dependent proteolysis in skeletal muscles of tumor-bearing rats. *Cancer Res* **54**: 5568–5573
Tessitore L, Bonelli G and Baccino FM (1987) Early development of protein metabolic perturbations in the liver and skeletal muscle of tumour-bearing rats. *Biochem J* **241**: 153–159
Tessitore L, Costelli P and Baccino FM (1993a) Humoral mediation for cancer cachexia in tumour-bearing rats. *Br J Cancer* **67**: 15–23
Tessitore L, Costelli P, Bonetti G and Baccino FM (1993b) Cancer cachexia, malnutrition, and tissue protein turnover in experimental animals. *Arch Biochem Biophys* **306**: 52–58
Tisdale MJ (1997) Biology of cachexia. *J Natl Cancer Inst* **89**: 1763–1773
Vezzoli G, Elli AA, Tripodi G, Bianchi G and Carafoli E (1985) Calcium ATPase in erythrocytes of spontaneously hypertensive rats of the Milan strain. *J Hypertens* **3**: 645–648
Watt F and Molloy PL (1993) Specific cleavage of transcription factors by the thiol protease, m-calpain. *Nucleic Acid Res* **21**: 5092–6000
Williams A, Sun X, Fisher JE and Hasselgren PO (1999a) The expression of genes in the ubiquitin-proteasome proteolytic pathway is increased in skeletal muscle from patients with cancer. *Surgery* **126**: 744–750
Williams AB, Decourten-Myers GM, Fischer JE, Luo G, Sun X and Hasselgren PO (1999b) Sepsis stimulates release of myofilaments in skeletal muscle by a calcium-dependent mechanism. *FASEB J* **1**: 1435–1443
Yoshida Y, Shiga T and Imai S (1990) Degradation of sarcoplasmic reticulum calcium-pumping ATPase in ischemic-reperfused myocardium: role of calcium-activated neutral protease. *Basic Res Cardiol* **85**: 495–507

 *© 2001 Cancer Research Campaign*

## References

[PDF_00412] Baracos V. E., DeVivo C., Hoyle D. H., Goldberg A. L. (1995). Activation of the ATP-ubiquitin-proteasome pathway in skeletal muscle of cachectic rats bearing a hepatoma.. Am J Physiol.

[PDF_00415] Beck S. A., Smith K. L., Tisdale M. J. (1991). Anticachectic and antitumor effect of eicosapentaenoic acid and its effect on protein turnover.. Cancer Res.

[PDF_00418] Bick R. J., Liao J. P., King T. W., LeMaistre A., McMillin J. B., Buja L. M. (1997). Temporal effects of cytokines on neonatal cardiac myocyte Ca2+ transients and adenylate cyclase activity.. Am J Physiol.

[PDF_00421] Busquets S., García-Martínez C., Alvarez B., Carbó N., López-Soriano F. J., Argilés J. M. (2000). Calpain-3 gene expression is decreased during experimental cancer cachexia.. Biochim Biophys Acta.

[PDF_00427] Carafoli E., Molinari M. (1998). Calpain: a protease in search of a function?. Biochem Biophys Res Commun.

[PDF_00429] Costelli P., Baccino F. M. (2000). Cancer cachexia: from experimental models to patient management.. Curr Opin Clin Nutr Metab Care.

[PDF_00431] Costelli P., Carbó N., Tessitore L., Bagby G. J., Lopez-Soriano F. J., Argilés J. M., Baccino F. M. (1993). Tumor necrosis factor-alpha mediates changes in tissue protein turnover in a rat cancer cachexia model.. J Clin Invest.

[PDF_00434] Das K. C., Lewis-Molock Y., White C. W. (1995). Activation of NF-kappa B and elevation of MnSOD gene expression by thiol reducing agents in lung adenocarcinoma (A549) cells.. Am J Physiol.

[PDF_00451] Furukawa K., Mattson M. P. (1998). The transcription factor NF-kappaB mediates increases in calcium currents and decreases in NMDA- and AMPA/kainate-induced currents induced by tumor necrosis factor-alpha in hippocampal neurons.. J Neurochem.

[PDF_00455] Hirai S., Kawasaki H., Yaniv M., Suzuki K. (1991). Degradation of transcription factors, c-Jun and c-Fos, by calpain.. FEBS Lett.

[PDF_00457] Jones P. L., Ping D., Boss J. M. (1997). Tumor necrosis factor alpha and interleukin-1beta regulate the murine manganese superoxide dismutase gene through a complex intronic enhancer involving C/EBP-beta and NF-kappaB.. Mol Cell Biol.

[PDF_00464] Kien C. L., Camitta B. M. (1987). Close association of accelerated rates of whole body protein turnover (synthesis and breakdown) and energy expenditure in children with newly diagnosed acute lymphocytic leukemia.. JPEN J Parenter Enteral Nutr.

[PDF_00461] Kien C. L., Camitta B. M. (1983). Increased whole-body protein turnover in sick children with newly diagnosed leukemia or lymphoma.. Cancer Res.

[PDF_00470] Liu Z. Q., Kunimatsu M., Yang J. P., Ozaki Y., Sasaki M., Okamoto T. (1996). Proteolytic processing of nuclear factor kappa B by calpain in vitro.. FEBS Lett.

[PDF_00477] Llovera M., Carbó N., García-Martínez C., Costelli P., Tessitore L., Baccino F. M., Agell N., Bagby G. J., López-Soriano F. J., Argilés J. M. (1996). Anti-TNF treatment reverts increased muscle ubiquitin gene expression in tumour-bearing rats.. Biochem Biophys Res Commun.

[PDF_00473] Llovera M., Garcia-Martinez C., Agell N., Lopez-Soriano F. J., Argiles J. M. (1995). Muscle wasting associated with cancer cachexia is linked to an important activation of the ATP-dependent ubiquitin-mediated proteolysis.. Int J Cancer.

[PDF_00481] Lundholm K., Ekman L., Karlberg I., Edström S., Scherstén T. (1980). Comparison of hepatic cathepsin D activity in response to tumor growth and to caloric restriction in mice.. Cancer Res.

[PDF_00484] Melloni E., Pontremoli S., Michetti M., Sacco O., Sparatore B., Horecker B. L. (1986). The involvement of calpain in the activation of protein kinase C in neutrophils stimulated by phorbol myristic acid.. J Biol Chem.

[PDF_00487] Melville S., McNurlan M. A., Calder A. G., Garlick P. J. (1990). Increased protein turnover despite normal energy metabolism and responses to feeding in patients with lung cancer.. Cancer Res.

[PDF_00490] Nath R., Raser K. J., Stafford D., Hajimohammadreza I., Posner A., Allen H., Talanian R. V., Yuen P., Gilbertsen R. B., Wang K. K. (1996). Non-erythroid alpha-spectrin breakdown by calpain and interleukin 1 beta-converting-enzyme-like protease(s) in apoptotic cells: contributory roles of both protease families in neuronal apoptosis.. Biochem J.

[PDF_00492] Niggli V., Penniston J. T., Carafoli E. (1979). Purification of the (Ca2+-Mg2+)-ATPase from human erythrocyte membranes using a calmodulin affinity column.. J Biol Chem.

[PDF_00495] Pontremoli S., Melloni E., Salamino F., Sparatore B., Viotti P., Michetti M., Duzzi L., Bianchi G. (1986). Decreased level of calpain inhibitor activity in red blood cells from Milan hypertensive rats.. Biochem Biophys Res Commun.

[PDF_00499] Pontremoli S., Melloni E., Sparatore B., Salamino F., Pontremoli R., Tizianello A., Barlassina C., Cusi D., Colombo R., Bianchi G. (1988). Erythrocyte deficiency in calpain inhibitor activity in essential hypertension.. Hypertension.

[PDF_00501] Pontremoli S., Melloni E., Viotti P. L., Michetti M., Salamino F., Horecker B. L. (1991). Identification of two calpastatin forms in rat skeletal muscle and their susceptibility to digestion by homologous calpains.. Arch Biochem Biophys.

[PDF_00505] Pontremoli S., Viotti P. L., Michetti M., Salamino F., Sparatore B., Melloni E. (1992). Modulation of inhibitory efficiency of rat skeletal muscle calpastatin by phosphorylation.. Biochem Biophys Res Commun.

[PDF_00508] Richard I., Broux O., Allamand V., Fougerousse F., Chiannilkulchai N., Bourg N., Brenguier L., Devaud C., Pasturaud P., Roudaut C. (1995). Mutations in the proteolytic enzyme calpain 3 cause limb-girdle muscular dystrophy type 2A.. Cell.

[PDF_00513] Salamino F., De Tullio R., Mengotti P., Viotti P. L., Melloni E., Pontremoli S. (1992). Different susceptibility of red cell membrane proteins to calpain degradation.. Arch Biochem Biophys.

[PDF_00516] Salamino F., De Tullio R., Michetti M., Mengotti P., Melloni E., Pontremoli S. (1994). Modulation of calpastatin specificity in rat tissues by reversible phosphorylation and dephosphorylation.. Biochem Biophys Res Commun.

[PDF_00520] Salamino F., Sparatore B., Melloni E., Michetti M., Viotti P. L., Pontremoli S., Carafoli E. (1994). The plasma membrane calcium pump is the preferred calpain substrate within the erythrocyte.. Cell Calcium.

[PDF_00523] Soeters P. B., Baracos V. E. (1999). Anabolic and catabolic mediators.. Curr Opin Clin Nutr Metab Care.

[PDF_00525] Temparis S., Asensi M., Taillandier D., Aurousseau E., Larbaud D., Obled A., Béchet D., Ferrara M., Estrela J. M., Attaix D. (1994). Increased ATP-ubiquitin-dependent proteolysis in skeletal muscles of tumor-bearing rats.. Cancer Res.

[PDF_00529] Tessitore L., Bonelli G., Baccino F. M. (1987). Early development of protein metabolic perturbations in the liver and skeletal muscle of tumour-bearing rats. A model system for cancer cachexia.. Biochem J.

[PDF_00532] Tessitore L., Costelli P., Baccino F. M. (1993). Humoral mediation for cachexia in tumour-bearing rats.. Br J Cancer.

[PDF_00534] Tessitore L., Costelli P., Bonetti G., Baccino F. M. (1993). Cancer cachexia, malnutrition, and tissue protein turnover in experimental animals.. Arch Biochem Biophys.

[PDF_00537] Tisdale M. J. (1997). Biology of cachexia.. J Natl Cancer Inst.

[PDF_00538] Vezzoli G., Elli A. A., Tripodi G., Bianchi G., Carafoli E. (1985). Calcium ATPase in erythrocytes of spontaneously hypertensive rats of the Milan strain.. J Hypertens.

[PDF_00541] Watt F., Molloy P. L. (1993). Specific cleavage of transcription factors by the thiol protease, m-calpain.. Nucleic Acids Res.

[PDF_00546] Williams A. B., Decourten-Myers G. M., Fischer J. E., Luo G., Sun X., Hasselgren P. O. (1999). Sepsis stimulates release of myofilaments in skeletal muscle by a calcium-dependent mechanism.. FASEB J.

[PDF_00543] Williams A., Sun X., Fischer J. E., Hasselgren P. O. (1999). The expression of genes in the ubiquitin-proteasome proteolytic pathway is increased in skeletal muscle from patients with cancer.. Surgery.

[PDF_00549] Yoshida Y., Shiga T., Imai S. (1990). Degradation of sarcoplasmic reticulum calcium-pumping ATPase in ischemic-reperfused myocardium: role of calcium-activated neutral protease.. Basic Res Cardiol.

